# Rational consideration of *Akkermansia muciniphila* targeting intestinal health: advantages and challenges

**DOI:** 10.1038/s41522-022-00338-4

**Published:** 2022-10-17

**Authors:** Yuheng Luo, Cong Lan, Hua Li, Qingyuan Ouyang, Fanli Kong, Aimin Wu, Zhihua Ren, Gang Tian, Jingyi Cai, Bing Yu, Jun He, André-Denis G. Wright

**Affiliations:** 1 Animal Nutrition Institute, Sichuan Agricultural University, Key Laboratory for Animal Disease-Resistance Nutrition of Ministry of Education of China, Key Laboratory for Animal Disease-Resistance Nutrition and Feed of Ministry of Agriculture of China, Key laboratory of Animal Disease-resistant Nutrition of Sichuan Province, Chengdu, 611130 China; 2 College of Animal Science and Technology, Sichuan Agricultural University, Chengdu, 611130 China; 3 College of Life Science, Sichuan Agricultural University, Ya’an, Sichuan China; 4 College of Veterinary Medicine, Sichuan Province Key Laboratory of Animal Disease and Human Health, Key Laboratory of Environmental Hazard and Human Health of Sichuan Province, Sichuan Agricultural University, Chengdu, 611130 China; 5 Department of Microbiology and Plant Biology, University of Oklahoma, 660 Parrington Oval, Norman, Oklahoma 73019 USA

**Keywords:** Applied microbiology, Symbiosis

## Abstract

As one of the promising next-generation probiotics (NGPs), *Akkermansia muciniphila*, a well-known mucin-degrading bacterium, has been proven to be closely related to the metabolic diseases of its human host. However, the role of *A. muciniphila* in the host’s intestinal health remains ambiguous. Here, we comprehensively summarize and discuss the characteristics, the distribution, and the colonization of *A. muciniphila* in the human gastrointestinal tract (GIT). We propose that the application of *A. muciniphila* as a biomarker for longevity, for diagnostics and prognostics of intestinal diseases, or for intestinal health should be cautiously considered. Precise dietary regulation can mediate the treatment of intestinal diseases by altering the abundance of *A. muciniphila*. Although the beneficial role of *A. muciniphila* and its component in intestinal inflammation has been discovered, in gnotobiotic mice with specific gut microbiota, certain genotype, and colorectal cancer, or in animal models infected with a specific pathogen, *A. muciniphila* may be related to the occurrence and development of intestinal diseases. Genomic analysis, emphasizing the strain-level phylogenetic differences of *A. muciniphila*, indicates that a clear description and discussion of each strain is critical before its practical application. Our review provides much needed insight for the precise application of *A. muciniphila*.

## Introduction

As a mucin utilizing specialist^1^, *Akkermansia muciniphila* has been highly considered as one of the next-generation probiotics (NGPs) and is regarded to play an important role in the maintenance of the intestinal epithelial barrier. A typical cycle of intestinal inflammation is driven by abnormal interactions among genetic risk factors, environmental triggers (microbiota), modifiers, and the host’s immune system^2^. *Akkermansia muciniphila* widely exists in the GIT of multiple animals including humans, mice^3^, cattle^4^, guinea pigs^5^, swine^6^, rabbits^7^, ostriches^8^ and chickens^9^. In infants and healthy adults, *A. muciniphila* can account for 1~3% of total fecal cells^10^, during which the excessive degradation of mucin allows pathogens to invade the sloughed intestinal mucosa^11^. In such cases, supplement with adequate numbers of *A. muciniphila*, or heat-killed *A. muciniphila* may safely improve the intestinal barrier in obese humans^12^ and mice fed high-fat diets^13,14^. However, an excessive enrichment of *A. muciniphila* in mice with a specific intestinal environment may lead to the aggravation of intestinal inflammation caused by epithelial barrier damage^15–17^. Although the effect of *A. muciniphila* on intestinal inflammation has been gradually studied, how it works is still unclear. Meanwhile, factors including host, intestinal segmentation, age, intestinal disease, and diet, affecting the distribution of *A. muciniphila* in the GIT and how *A. muciniphila* interacts with the host to maintain intestinal health is mainly unknown. In this review, we bring together the latest research to comprehensively discuss the potential of *A. muciniphila* as a NGP to intervene in the intestinal homeostasis in humans and animals.

## The characteristics and safety of *A. muciniphila* in the GIT

Belonging to the phylum *Verrucomicrobia*, *A. muciniphila* has been described as an oval-shaped, non-mobile, Gram-negative, non-spore forming, and strictly anaerobic bacterium. However, more than 90% number of *A. muciniphila* ATCC BAA-835 can survive in 95% oxygen and 5% CO_2_ for 1 h^18^. Different strains and phylogroups of *A. muciniphila* differ in their sensitivity to oxygen^19^, and most of the known *A. muciniphila* strains can utilize mucin as the sole carbon and nitrogen sources. The bacterium can grow on the Brain Heart Infusion (BHI) and Columbia medium, and mucin-derived monosaccharides, such as fucose, galactose, and *N*-acetylglucosamine, can also be used by *A. muciniphila* as growth substrates^20^.

The complete genome of type strain, *A. muciniphila* ATCC BAA-835, is 2,664,102 bp long, and has 2,176 predicted protein-coding genes, which suggest it can metabolize different kinds of carbohydrates and mucin^21^. Phylogenetic analysis of *A. muciniphila* classified it into three^22^ or four^23^ species-level phylogroups. *Akkermansia muciniphila* MucT strain is resistant to several antibiotics, such as chloramphenicol, clindamycin, streptomycin, erythromycin, vancomycin, and metronidazole^24,25^. The MucT strain is also abundantly colonized in the GIT of individuals treated with broad-spectrum antibiotics^25^, which may be due to the fact that *A. muciniphila* is an open-pangenome microorganism that can continually acquire genes from other bacteria via lateral gene transfer^22^.

Nowadays, *A. muciniphila* is widely studied as a promising probiotic to improve metabolic syndrome and obesity. However, its safety and toxicity are a growing concern. Long-term oral high-dosage of *A. muciniphila*, or pasteurized *A. muciniphila* (10^10^ bacteria per day), are safe and well tolerated in overweight and obese individuals^12^. The bacterial reverse mutation, in vitro mammalian cell micronucleus test, and a subchronic toxicity test (lasting 90 days in rat), show that pasteurized *A. muciniphila* has no-adverse effects^26^. Recently, pasteurized *A. muciniphila* has been recognized as a new food by the European Union^27^. Based on these findings and policies, the utilization of *A. muciniphila* in metabolic syndrome and in healthy individuals may be safe. However, whether *A. muciniphila* treatment is safe, when intestinal diseases occur, still needs to be confirmed.

## The colonization and abundance of *A. muciniphila* in GIT

### Location-dependent colonization of *A. muciniphila* in the GIT

**Fig. 1:**
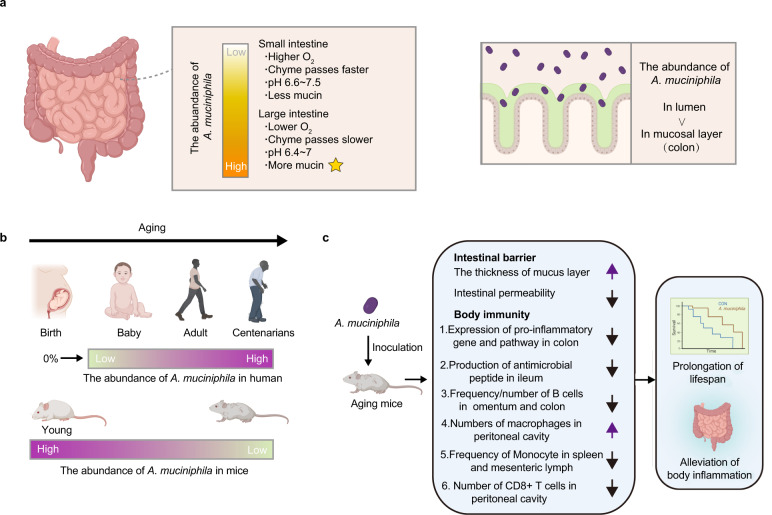
The abundance and role of *A. muciniphila* with spatial and temporal change in the GIT. **a** The distribution of *A. muciniphila* along the GIT (small and large intestine) and in the lumen and mucosal layer. **b** The schematic diagram of *A. muciniphila* abundance changing with age in the human and mouse GIT. **c** The mechanisms of *A. muciniphila* ameliorating aging in mice. All figures are created with Biorender.com.

The abundance of *A. muciniphila* in the GIT seems to be location-dependent. Bacteria, belonging to the phylum *Verrucomicrobia*, not specified to *Akkermansia*, can be detected in human duodenal biopsies (0.0688%) and mucus (0.0387%)^28^. *Akkermansia* species, with an average relative abundance of 0.01%, are also found in the jejunal content of humans^29^, but the abundance of *Verrucomicrobia-related* bacteria can make up 5% of the bacteria density in the distal ileum of humans, and as much as 6% and 9% in the ascending colon and rectum mucosal biopsies, respectively^30^. Compared to the small intestine, the passage time of chyme is much longer (9–46 h)^31^ and the mucosal layer is thicker in the large intestine, which is presumed to provide multiple substrates for *A. muciniphila*^32^. As a mucin-degrading bacterium, *A. muciniphila* is abundantly found in the mucin-rich intra-intestinal location^33^, for which it is positively correlated with the concentration of mucin^34^. For instance, inoculated *A. muciniphila* is found to efficiently colonize (13.08% of total microbes) in the caecum of chickens^9^. In humans, there are approximate 1.45 × 10^4^ cells of *A. muciniphila* per gram of ascending, or sigmoid colonic mucosal biopsies^35^. Moreover, the proportion of *Akkermanisa* in the lumen (0.57%) is found higher than that in colonic mucosa of healthy individuals (0.21%)^36^. The pH value may be another factor affecting the distribution of *A. muciniphila* in different intestinal segments. The pH value of the small and large intestine is 6.6~7.5 and 6.4~7.0, respectively^37^ (Fig. 1a). Using a model of the human digestive system, Simulator of the Human Intestinal Microbial Ecosystem (SHIME), when the pH value of the distal colon is 6.6~6.9, the abundance of *A. muciniphila* is at its highest^38^.

### Aging-dependent colonization of *A. muciniphila* in GIT

Tracking the fecal bacterial composition in 98 infants from birth to 12 months old shows a gradual increase (0% to 0.57%) in the relative abundance of *A. muciniphila*^39^. Similar increase (0.14% to 4.25%) is found in children aged from 1 to 4 years^40^. In addition, the abundance of *A. muciniphila* is high in long-living Chinese people (≥90 years old)^41^. Moreover, the abundance of *A. muciniphila* is especially higher in the gut of older populations aged from 105 to 109 years old, compared to other age groups^42^ (Fig. 1b). These findings^43–46^ (Table 1) give rise to the consideration of exogenous *A. muciniphila* inoculation to alleviate the negative effects of aging^47,48^ (Fig. 1c). However, opposite results are found in studies using rodents. The abundance of *Akkermansia* appears to be decreaed in aged mice or rats^3,49,50^. Although *A. muciniphila* may be a potential biomarker of longevity in humans, mice may not be a natural research model to study this relationship in humans.

**Table 1:** The abundance of *A. mucinihlia* varies with age.

Author/Year	Volunteers	Geographic area	Method	Main Findings
Elena Biagi et al. 2016^42^	22–48 years: *n* = 15 65–75 years: *n* = 15 99–104 years: *n* = 15 105–109 years: *n* = 24	Emilia Romagna and surrounding area, Italy	16S rRNA gene sequencing	The relative abundance of *A. muciniphila* is increased in 105–109 years old humans.
Fanli Kong et al. 2016^41^	24–64 years: *n* = 47 65–83 years: *n* = 54 90–102 years: *n* = 67	Dujiangyan and Ya’an, Sichuan, China	16S rRNA gene sequencing	Relative abundance of *Akkermansia* OTUs in 90–102 years old humans is higher than that in younger people.
Simone Rampelli et al. 2020^46^	22–48 years: *n* = 11 65–75 years: *n* = 13 99–104 years: *n* = 15 105–109 years: *n* = 23	Emilia Romagna, Italy	Shotgun sequencing	Compared with younger individuals, long-lived humans show a significantly increase of *A. muciniphila*.
Nuria Salazar et al. 2019^44^	<50 years: *n* = 49 50–65 years: *n* = 58 66–80 years: *n* = 19 >80 years: *n* = 27	The central area of the Asturias Region, northern Spain	Real-time PCR	The counts of *Akkermansia* in older humans (>80 years) is higher than that in younger population.
Bong-Soo Kim et al. 2019^45^	26–43 years: 9 67–79 years: 17 95–108 years: 30	The neighboring counties of Gurye, Gokseong, Sunchang, and Damyang, located in the southwestern part of Korea	16S rRNA gene sequencing	Centenarians have higher levels of *Akkermansia* in their gut.

## Factors influencing the colonization and abundance of *A. muciniphila* in the GIT

### The abundance of *A. muciniphila* related to different intestinal diseases

Inflammatory bowel disease (IBD), including Crohn’s disease (CD) and ulcerative colitis (UC), is a known risk factor for the development of colorectal cancer (CRC), like colitis-associated colorectal cancer (CAC)^51^, the third leading cause of cancer-related death in humans^52^. The number of *A. muciniphila* in healthy individuals is higher than that in IBD patients^53,54^ (Supplementary Table 1), especially in the hindgut^55^. The relative abundance of *A. muciniphila* can be as high as 2.9% in healthy populations, but is found to sharply decline in noninflamed UC (0.03%), inflamed UC (0.02%), noninflamed CD (0.62%), and inflamed CD (0.20%) patients^56^. Moreover, *A. muciniphila* are more abundant in CD patients than in UC patients^54,56^.

However, the higher abundance of *A. muciniphila* may not be negatively correlated with IBD. A surprising result shows in both CRC patients and CRC mice, the abundance of *A. muciniphila* is higher than that in healthy people^57,58^ (Supplementary Table 1). Moreover, *A. muciniphila* is enriched in the early stage of CRC^59^. The abundance of *A. muciniphila* may also be increased by pathogenic infection^60,61^ (Supplementary Table 1).

### Diet and lifestyle can regulate the abundance of *A. muciniphila*

Diet is an important factor that cannot be ignored to shape the gut microbiota^62,63^. We summarized previous studies and focused on the relationship between the abundance of *A. muciniphila* and dietary ingredients, which are associated with host health and intestinal diseases. The high-concentration of cellulose in the diet can relieve the inflammation of dextran sodium sulfate (DSS)-induced mice, while increasing the abundance of *A. muciniphila*^64^. A diet enriched with rye bran and wheat aleurone is reported to increase the relative abundance of *Akkermansia* in C57BL/6 J mice, accompanied by changes in glycine betaine metabolism^65^. Both sugarcane bagasse, a water-soluble fiber, and xylo-oligosaccharide can also increase the abundance of *Akkermansia* in Fischer 344 rats^66^. Milk and its products, for example, breast milk can promote the growth of *A. muciniphila* in mice transplanted with microbiota from infant^67^, which may be triggered by galacto-*N*-biose^68^. Another study revealed that the consumption of cheese is negatively associated with the abundance of *A. muciniphila*^69^. The increase of *A. muciniphila* by dietary supplement of polyphenol containing grape proanthocyanidin, chlorogenic acid, and resveratrol is accompanied by the improvement of metabolic profile and anti-inflammatory activities of host, especially in mice with DSS-induced colitis^70–72^. Interestingly, grape proanthocyanidin may indirectly induce the intestinal bloom of *A. muciniphila*, in vivo, in mice, but shows no effect on the quantity of *A. muciniphila* in vitro^70^. Probiotics, such as *Lactobacillus fermentum* and *Bacillus subtilis*, can alleviate DSS-induced colits in mice and increase the abundance of *Akkermansia*^73,74^. In contrast, other probiotics, such as *Bifidobacteria adolescentis*, is found to inhibit the excessive growth of *A. muciniphila* during the therapy of DSS-induced chronic colitis^75^. Similarly, *Pediococcus pentosaceus* and *Lactobacillus coryniformis* can ameliorate CRC in mice via regulating gut microbiota, including increasing the abundance of *A. muciniphila*^76,77^. Particular dietary patterns, such as low-calorie diet, ketogenic diet, and fasting, are reported to increase the abundance of *A. muciniphila* in healthy individuals, or IBD patients^78–81^. It is worth noting that gut microbial composition can be influenced by many factors, especially stool consistency and fecal transit time, which are closely connected with the abundance of *A. muciniphila*^82,83^. To summarize, *A. muciniphila* may participate in the effect of diet on IBD, but whether the change of *A. muciniphila* abundance is the cause, or result, remains to be determined.

## *A. muciniphila* and intestinal homeostasis of host

### *A. muciniphila* and the intestinal physical barrier of host

Live *A. muciniphila* bacteria have been repeatedly confirmed to be related to the improvement of the intestinal barrier. Oral gavage with live *A. muciniphila* can increase the expression of tight junction proteins (TJs), such as zonula occludens (ZO-1) and occludin, in DSS-induced mice^84^. In vitro, active *A. muciniphila* bacteria are also found to increase the transepithelial electrical resistance (TER), a recognized parameter to reflect the cell integrity of the cell membrane^85^ of cocultured Caco-2 cells after 24 or 48 h^18,86^. Particularly, some cellular components of *A. muciniphila* have also been shown to improve the intestinal permeability. One of them is extracellular vesicles (AmEVs), the lipid bilayer secreted by *A. muciniphila*. Compared to obese mice induced by high-fat diet, or lipopolysaccharide (LPS)-induced Caco-2 cell, the expression of occludin, ZO-1, and claudin-5 is enhanced (in vivo and in vitro) by activating the adenosine monophosphate (AMP)-activated protein kinase (AMPK) pathway in a dose-dependent manner with oral administration of 10 μg AmEVs^87^. Moreover, after pasteurization^88^, a stable outer membrane of *A. muciniphila*, Amuc_1100, has been shown to increase the TER in vitro^86^ and the expression of TJ genes in the small intestine of obese mice induced by high-fat diet in vivo^14^. Amuc-1100 belongs to a gene cluster related to the formation of pilus^86^ and was recently used in mice with metabolic and intestinal diseases^14,88^.

**Fig. 2:**
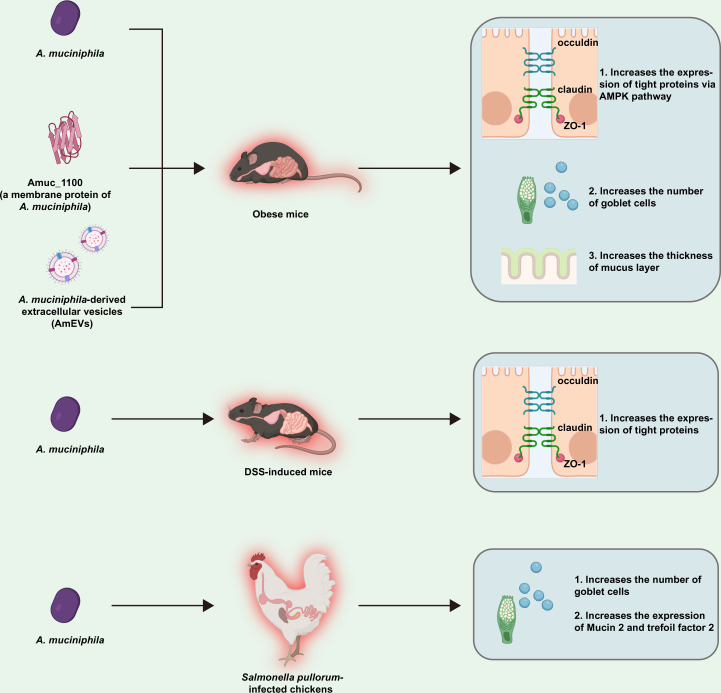
The possible mechanisms of *A. muciniphila* regulating intestinal barrier summarized according to existing references. All figures are created with Biorender.com.

As a mucin-specialist, the abundance of *A. muciniphila* is closely related to the thickness of the intestinal mucosa. A similar result is found in *Apoe*^*−/−*^ mice fed western-diet^89^. Goblet cells, a specialized epithelial cell that secretes mucins, have attracted much attention because of their important role in maintaining the integrity of the inner mucus layer^90^. A gavage with 1.0 × 10^8^ CFU/day of *A. muciniphila* (DSM 22959) can increase the density of goblet cells in the ileum of mice with a long-term feeding of high-fat diet^91^. Similarly, *A. muciniphila* bacteria are believed to increase the number of goblet cells and up-regulate the expression of Mucin 2 (MUC2) and trefoil factor 2 (Tff2) in *Salmonella pullorum*-infected chickens^92^. A genome-wide association study (GWAS) based on 288 pigs revealed a correlation between the relative abundance of *A. muciniphila* and a gene encoding carbohydrate sulfotransferase 12^93^, a required gene for the biosynthesis of glycosaminoglycan and the formation of mucin^94,95^. It should be highlighted that the genome of *A. muciniphila* (ATCC BAA-835) lacks mucus-binding domains^21^, which is verified by an in vitro study that *A. muciniphila* can barely adhere to the mucus^18^. These results describe the protective effect of *A. muciniphila* on intestinal mucosa, which may be related to the increase of goblet cells (Fig. 2).

### *A. muciniphila* and the intestinal immunity of the host

**Fig. 3:**
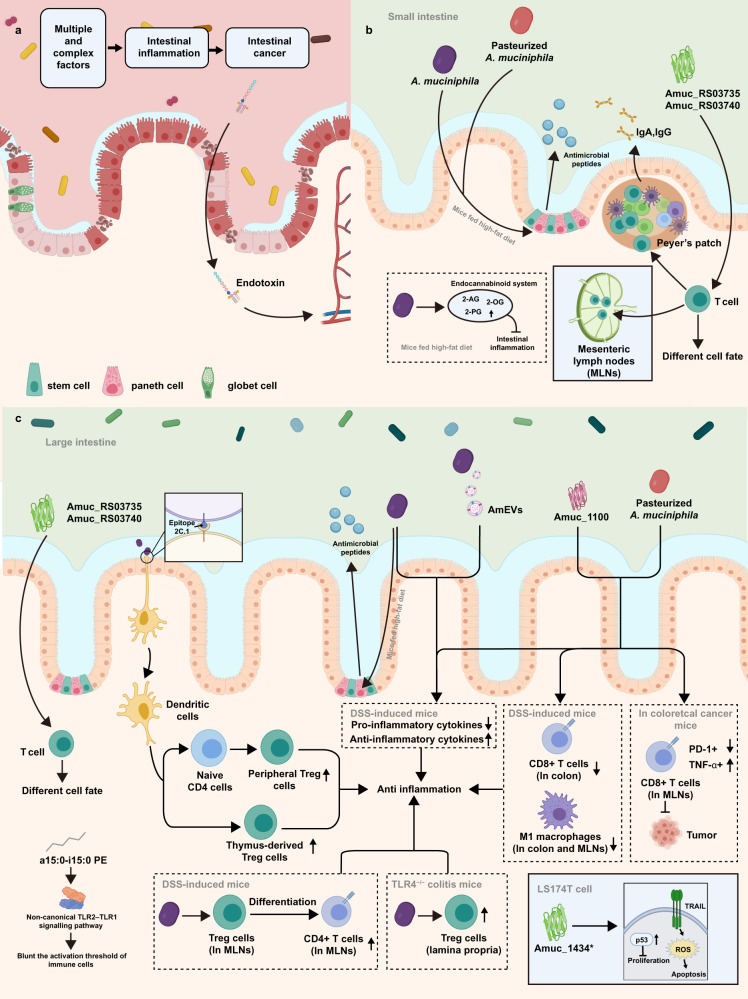
The possible mechanisms of *A. muciniphila* regulating intestinal immunity in host with intestinal inflammation and colon cancer. All figures are created with Biorender.com.

The intestinal inflammation involves the complex interaction of host genes, host immunity, microbiota and environmental factors (Fig. 3a). As a mucin-degrader in the gut, *A*. *muciniphila* can easily induce the immune response of the host due to its frequent communication with intestinal epithelial cells (IECs) (Fig. 3b and c). For instance, *A. muciniphila* increases the expression of genes encoding 2-oleoylglycerol, 2-arachidonoylglycerol and 2-palmitoylglycerol in the ileum of mice^13^, which are associated with the endocannabinoid system involving intestinal homeostasis and improved intestinal barriers^96^. When *A. muciniphila* is present in the intestine of specified pathogen free (SPF) mice, T cells response to *A. muciniphila* are localized to the Peyer’s patches (PPs), large intestine, small intestine lamina propria and mesenteric lymph nodes (mLNs), which is regulated by the outer membrane proteins Amuc_RS03735 and Amuc_RS03740^97^. In mice with oral treatment of live *A. muciniphila*, the differentiation of peripheral regulatory T cells (pTregs), the proliferation of residual thymus-derived Tregs (tTregs) in the colon (which reprogramed by epitope 2 C.1 from *A. muciniphila*^98^), and the differentiation of Foxp3^+^ Treg from CD4^+^ T cells in MLNs are found to be promoted^99^. *Akkermansia muciniphila* is also found to be positively correlated with TLR4 receptor and against TLR4^−/−^ induced colitis in mice by increasing the proportion of RORγt^+^ Treg cells that enhances the immune response^100^. Whereas, in altered Schaedler flora (ASF) mice, the treatment of *A. muciniphila* specifically impacted the number of T follicular helper (T_FH_) cells only in the Peyer’s patches (PPs)^97^. As the T_FH_ cells are important for the secretion of immunoglobulins (e.g. IgA), the variation in the quantity of these cells may help to slow down the advanced-stage intestinal inflammation^101^. Besides the proliferation, the development of immune cells is also involved in the abundance of *A. muciniphila*. In addition, both pasteurised *A. muciniphila* and Amuc_1100 can decrease the colonic infiltration of CD8^+^ cytotoxic T lymphocytes (CTLs), which aggravates colitis by mediating the production of cytokines^102,103^, and can suppress the proliferation of proinflammatory CD16/32^+^ macrophages in the MLNs and decrease the mRNA level of pro-inflammatory cytokines in mice with DSS-induced colitis^88^. In a mice model with CRC, pasteurised *A. muciniphila* and Amuc_1100 increased the activation of CTLs in the MLN and the proportion of tumor necrosis factor-alpha (TNF-α)^+^ CTLs to promote the apoptosis of tumor cells. Meanwhile, the proportion of PD-1^+^ CTLs in MLN can be decreased to suppress the growth of tumor^88^. Another protein of *A. muciniphila*, Amuc_1434, an aspartic protease can degrade MUC2 in vitro^104^, can inhibit the proliferation of LS174T cells and block the G0/G1 phase of cell cycle of LS174T cells by increasing the expression of tumor protein 53 (p53) in vitro^105^. Further, Amuc_1434* treatment promotes the apoptosis of LS174T cells and increases the level of mitochondrial reactive oxygen species (ROS) by upregulating tumor-necrosis-factor-related apoptosis-inducing ligand (TRAIL)^105^. The concentration of inflammatory cytokines can be used as an important indicator to assess the severity of intestinal inflammation. The pretreatment of *A. muciniphila* was found to suppress the expression of pro-inflammatory cytokines, such as interferon gamma (IFN-γ), interleukin-17 (IL-17), TNF-α, interleukin-1beta (IL-1β) and nitric oxide synthase 2 (NOS2), in the colon of mice with DSS-induced colitis^106^. Similarly, the mRNA level of pro-inflammatory cytokines, TNF-α, IFN-γ, IL-1β, IL-6, IL-18 and IL-33, in the colon of mice with DSS-induced colitis can be also decreased by the treatment of pasteurised *A. muciniphila* (1.5×10^8^ CFU) or 3 µg of Amuc_1100^88^. In vitro, the level of IL-6 in colonic epithelial cells (CT26), challenged by *E. coli*-derived extracellular vesicle, can be reduced by the pre-treatment of AmEVs in a dose-dependent manner^107^. Adiacyl phosphatidylethanolamine, with two branched chains (a15:0-i15:0 PE), isolated from *A. muciniphila* can cause the release of specific inflammatory cytokines by acting on the non-classical TLR2-TLR1 heterodimer, and at low doses, can blunt the activation threshold of immune cells^108^.

The production of antigen-specific T cell-dependent IgA and IgG1 in the serum of ASF mice is reported to be induced by acquiring *A. muciniphila* vertically from mothers^97^. Live *A. muciniphila* bacteria markedly increases the expression of regenerating islet derived 3-gamma (Reg3g)^13^, a lectin protecting the intestinal mucosa against the invasion of pathogens^109^, in the colon of mice fed high-fat diet. In contrast, both live and pasteurized *A. muciniphila* improved the expression of lysozyme C-1 (Lyz1) in the small intestine of obese mice induced by high-fat diet^14^ (Fig. 3b and c).

### The interaction between *A. muciniphila* and the intestinal epithelium

A few studies suggest a direct effect of *A. muciniphila* on IECs. A linear discriminate analysis clearly shows the enrichment of *A. muciniphila* in the early regenerative mucosa of mice. The intrarectal administration of active *A. muciniphila* remarkably facilitate the closure of injured mucosa (from 43.7% to 74.14%) in mice by promoting the proliferation and migration of intestinal stem cells (ISCs) and accelerating the regeneration of the wound in SK-CO15 monolayers in vitro^110^. This requires the participation of formyl peptide receptor 1 (FPR1) and neutrophilic NADPH oxidase (NOX1) to increase ROS in the wound edge and the phosphorylation of extracellular-signal-regulated kinase (ERK) in colonic epithelial cells. In addition, the gavage of AmEVs isolated from *A. muciniphila* can alleviate dysplasia in C57BL/6 mice induced by 2% DSS^107^. Amuc_1100 (3 μg) can also relieve the shortening of colon and the histological injuries in the proximal colon in mice with DSS-induced colitis^88^, indicating an alleviation or even the repair of injured intestinal epithelium by *A. muciniphila*, or its derivatives.

The steady renewal of the IECs is fueled by ISCs lying at the basilar part of crypts^111^, which is particularly important in case of disrupted intestinal homeostasis. The colonization of *A*. *muciniphila* in the chicken colon is found to regulate the proliferation of ISCs though the classical Wnt/β-catenin signaling pathway^92^. In addition, *A*. *muciniphila* can closely bind to laminin^18^, one of the important components of extracellular matrix which can regulate the migration, differentiation and anti-inflammatory responses of IECs^112–114^. A GWAS based study showed a strong connection between laminin β1 chain encoding gene and the susceptibility of UC^115^, and showed the laminin γ1 chain encoding gene as a susceptible locus of IBD^116^. However, the interaction between *A. muciniphila* and laminin is still poorly understood. Therefore, as a bacterium that is directly communicated with intestinal mucosa, *A*. *muciniphila* displays an intervention in the proliferation and/or differentiation of IECs and ISCs, which represents a very complex cross-talk to be further discussed.

### Relationship between *A. muciniphila* and other intestinal bacteria during intestinal inflammation

Although *A. muciniphila* is found to negatively correlate with total mucin-degrading bacteria, its decreased number may result in the proliferation of mucin-associated bacteria when intestinal inflammation occurs^56^. This can reduce the degradation of mucus and maintain a relatively stable intestinal barrier^56^. Several studies provide direct evidence for such interaction between *A. muciniphila* and other mucosa-associated bacteria. When cocultured with mucolytic bacteria like *Bacteroides vulgatus*, *Ruminococcus gnavus*, or *Ruminococcus torques*, in a defined medium with MUC2 as sole carbon source, the growth of *A. muciniphila* is inhibited while the growth of other bacteria is promoted^56,117^. On the other hand, *A. muciniphila* may influence the intestinal microbiota by regulating the intestinal immunity of the host^13,118^. *Akkermansia muciniphila* treatment accelerates the normalization of the microbial community in mice with colitis, and reverses the decreased ratio of Firmicutes/Bacteroidetes bacteria in the cecum caused by high-fat diet^119^. A correlation between the abundance of *A. muciniphila* and *Faecalibacterium prausnitzii* is also confirmed in the feces of CD patients^120^. Moreover, six genera (*Prevotella*, *Sutterella*, *Klebsiella*, *Dorea*, *Parabacteroides*, and *Akkermansia*) are found to flourish in CD patients with remission^121^. Furthermore, both *A. muciniphila*-*F. prausnitzii*, and *A. muciniphila*-*Bacteroides thetaiotaomicron*, in IBD patients, are lower than in healthy individuals^54^, suggesting a relationship between mutualistic symbiosis of mucolytic bacteria and IBD.

## The negative effect of *A. muciniphila* in specific GIT environment

In several cases, *A. muciniphila* may have a negative impact on intestinal health (Table 2). Specifically, in a gnotobiotic C3H mouse model with eight bacterial species normally found in humans, the infection of *Salmonella typhimurium* with the pro-colonization of *A. muciniphila* makes the former a dominant bacterium in this limited microbiota accompanied by more severe intestinal inflammation^16^. Another study shows that *A. muciniphila* is able to induce colitis in specific-pathogen-free and germ-free *Il10*^*−/−*^ mice and its colonization is mediated by Nod-like receptor 6^17^. Low-fiber diet promotes expansion of *A. muciniphila* and other mucus-degrading bacteria in mice colonizing with a synthetic human gut microbiota, which promotes the degradation of the mucus layer and increases the colitis caused by *Citrobacter rodentium* infection^15^. In CRC mice transplanted with the fecal microbiota from CRC patients, *Akkermansia* bacteria are positively correlated with increased tumor burden^122^. In addition, gavage of *A. muciniphila* into intestine-specific *Apc* mutant mice (FabplCre; *Apc*^15lox/+^) aggravates the development of colorectal cancer by increasing the number of tumors^123^. In conclusion, *A. muciniphila* may be at risk of exacerbating pathogenic infections and inflammation of intestine, which is a common problem to be considered in mucin-degrading bacteria^124^.

**Table 2:** The negative effects of *A. muciniphila* on intestinal disease in some special cases.

Author/Year	Object	Model	Experimental design	Negative effect
Mahesh S. Desai et al.^15^	Mouse	Low-fiber diet and pathogen infection	Gnotobiotic mice are constructed with a synthetic gut microbiota from fully sequenced human gut bacteria, fed a fiber-deprivation diet (chronic or intermittent) and used *Citrobacter rodentium* to infect mice with two diet models to investigate the mechanistic connections between dietary fiber deficiency and microbiota composition, as well as the resulting effects on the mucus barrier.	Low-fiber diet promotes expansion and activity of mucus-degrading bacteria, such as *A. muciniphila*, which alleviates the degradation of the mucus layer and increases the susceptibility of pathogen-associated colitis.
Sergey S. Seregin et al.^17^	Mouse	Immune deficiency disorders associated with IBD	16 S rRNA sequencing is used to analyze the change of gut microbiota in *Il10*^*−/−*^ mice with spontaneous colitis and innate immune receptor NLRP6 deficiency, and oral gavage of screened strains is performed to investigate its effects in these mice.	1. The relative abundance of *A. muciniphila* is significantly increased in *Il10*^*−/−*^ *Nlrp6*^*−/−*^ mice. 2. *A. muciniphila* promotes colitis represented by the decreasing of body weight, as well as the increase of the colonic histological scores, weight of spleen, inflammation indication of colon, level of fecal Lcn-2, bacterial translocation to MLNs and pro-inflammatory mediators in the colons of both SPF *Il10*^*−/−*^ mice and germ-free *Il10*^*−/−*^ mice.
Héctor Argüello et al.^131^	Pig	*S. typhimurium* infection	16 S rRNA sequencing is used to analyze the composition of mucosa microbiome in the ileum of 28 days old pigs with *S. typhimurium* infection.	1. Genus *Akkermansia* increases within the mucosa of the *S. typhimurium* infected pigs. 2. Epithelial damage is positively correlated to taxa belonging to the phyla *Verrucomicrobia* such as *A. muciniphila*.
Bhanu Priya Ganesh et al.^16^	Mouse	*S. typhimurium* infection	Oral gavage of *A. muciniphila* followed by subsequently infection of *S. typhimurium* in gnotobiotic C3H mouse model with a background microbiota of eight bacterial species to research the impact of *A. muciniphila* on inflammatory and infectious symptoms.	1. After 5 days infection, *S. typhimurium* become the predominant species representing 94.03% of total bacteria in the cecum of mice co-colonized by *A. muciniphila* and *S. typhimurium*. 2. Co-colonization of *A. muciniphila* and *S. typhimurium* causes significantly higher histological scores and elevates the mRNA levels of pro-inflammatory cytokines, especially IFN-γ, IP-10, TNF-α, IL-12, IL-6, IL-17 in the cecum and colon of the infected mice. 3. The number of mucin-filled goblet cells, the thickness of mucus and mucus sulphation are significantly decreased by the co-colonization of *A. muciniphila* and *S. typhimurium*. 4. The existence of *A. muciniphila* may induce the deeper colonization of *S. typhimurium* in cecal tissue and encourages the recruitment of macrophages into the cecal lamella propria and submucosa.
Nielson T Baxter et al.^122^	Mouse	CRC	The fecal microbiota from three CRC patients and three healthy individuals are transplanted into germ-free mice, respectively. then, these mice are chemically induced to CRC resulting in different levels of tumorigenesis. The change of gut microbiome is investigated using 16 S rRNA sequencing and metagenomic analysis.	The taxa most strongly positively correlate with increased tumor burden are several Gram-negative species including *Akkermansia*.
Joseph P. Zackular et al.^132^	Mouse	CRC	The development of microbiome during the tumorigenesis in a mouse model with inflammation-driven colon cancer is investigated using 16 S rRNA sequencing.	1. Tumor-bearing mice show an enrichment in OTUs affiliated with members of *Akkermansia*. 2. The tumorigenesis in the colon of germ-free mice transplanted with the fecal microbiota from mice with tumor is increased.
Celia Dingemanse et al.^123^	Mouse	CRC	Shotgun metagenomic sequencing plus quantitative PCR is used to analyze the gut microbiota in intestine-specific conditional *Apc* mutant mice (FabplCre; *Apc*^15lox/+^) with large intestine tumor. Then, the FabplCre; *Apc*^15lox/+^ mice are treated with the identified specific bacteria by orally gavage to investigate their impact on the development of tumor.	1. Metagenomic sequencing shows that the genus *Akkermansia* is responsible for the overrepresentation in the conventional samples with more intestinal tumors. 2. The oral gavage of *A. muciniphila* to antibiotic-pretreated FabplCre; *Apc*^15lox/+^ mice significantly increases the number of intestinal tumors. 3. *A. muciniphila* significantly increases the thickness of intestinal mucus layer and the goblet cell ratio in FabplCre; *Apc*^15lox/+^ mice which may aggravate adenomatous in tumor-susceptive mice.

## The inspiration of precise application: strain-specific role of *A. muciniphila* on host intestinal health associated with its genetic and phenotypic properties

**Fig. 4:**
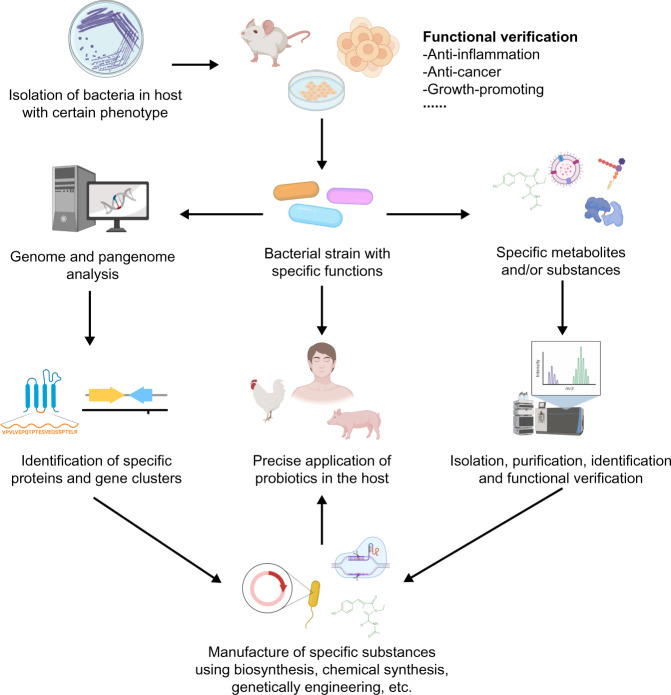
A schematic diagram of workflow on the precise application of NGP. All figures are created with Biorender.com.

The role of probiotics largely depends on the bacterial strains used, which is essential for their clinical effects^125^. Different bacterial strains have distinct genomic homology leading to discrepant function^126,127^, which makes it reasonable to consider the practical application of different strains. A total of 106 *A. muciniphila* metagenome-assembled genomes (MAGs) have been reconstructed based on the available metagenomic datasets of human, mouse and pig, which revealed three phylogroups of *A. muciniphila*, AmI, AmII and AmIII with different relative abundance^22^. Based on the whole-genome shotgun sequencing of 39 isolates of *A. muciniphila*, from human and mouse feces, three *A. muciniphila* phylogroups (AmI, AmII and AmIII) are identified and the functional annotation shows their distinct metabolic and functional features^22^. The comparative genomic analysis based on 35 metagenome-assembled genomes (MAGs) and 40 publicly available genomes further reveals at least four phylogroups of *A. muciniphila* (AmI to AmIV) and some strains in specific phylogroup have the genes and ability to vitamin B12 biosynthesis^23^. A study including genomic analysis and phenotypic test shows distinct characteristics of these phylogroups, including oxygen tolerance, cell adherence, the activation of toll-like receptor 2, sulfur acquisition and the colonization of the bacterium in GIT^19^. A large-scale metagenomic-based genomic analysis further confirms that the genomic difference may diversify the effect of *A. muciniphila* strains on host health^128,129^, and results of in vivo and in vitro studies support this hypothesis. In mice with chronic colitis, *A. muciniphila* strain ATCC 835 presents better anti-inflammatory properties than strain 139^99^. Of 11 human-derived *A. muciniphila* strains, only the supernatant from a culture of the AK32 strain can increase the size of small intestine-derived organoids in vitro^130^. It can be assumed that the function of different *A. muciniphila* strains may be various, possibly due to the diversity in their cellular components and metabolites, although most related studies focus on *A. muciniphila* ATCC 835. Moreover, function-specific component of different *A. muciniphila* strains, or their metabolites may be mass produced or recombined to investigate and reveal the effects and mechanism of *A. muciniphila* targeting diseases (Fig. 4). Based on the understanding of functional characterization of *A. muciniphila* strains, studies on the phenotypes of *A. muciniphila* in vitro and its effect on the host are required for the precise application of *A. muciniphila* in disease treatment.

In summary, regardless of host animal species, *A. muciniphila* is found to be more abundant in the hindgut. The abundance of *A. muciniphila* in the human GIT increases with age, which is contrary to that in mice. Types of intestinal diseases, dietary supplements, as well as other mucus-associated microbes can influence the abundance of *A. muciniphila*, but cautious consideration should be given to *A. muciniphila* as a biomarker for indicating an intestinal health risk. *Akkermansia muciniphila* may safety be administered in healthy individuals or those with metabolic syndrome (excess fat around the waist, high blood sugar, increased blood pressure, and abnormal cholesterol levels). *Akkermansia muciniphila* may also be beneficial to the maintenance of intestinal homeostasis of the host. However, in some cases, such as the lack of dietary fiber, pathogenic infection, or specific host genotypes, the accumulation of *A. muciniphila* in the GIT may exacerbate the damage of the intestinal epithelium, indicating that *A. muciniphila* may have a double-edged effect on the intestinal health of the host. In view of the strain-specific genome and phenotype of *A. muciniphila*, a clear description and discussion of each strain is critical before its practical application.

## Supplementary information

Supplementary Table1
