# Supplementary material for: Rational consideration of Akkermansia muciniphila targeting intestinal health: advantages and challenges
Source: NPJ Biofilms Microbiomes. 2022 Oct 17;8:81. doi: 10.1038/s41522-022-00338-4 (PMC9576740; doi:10.1038/s41522-022-00338-4)
Supplement: Supplementary file 1 — Supplementary Table1 [file 41522_2022_338_MOESM1_ESM.pdf]

Supplementary table 1. The abundance of *A. mucinihlia* in different intestinal disease

Disease	Host	The abundance of *A. mucinihlia*	reference
IBD	Human	-	[1–10]
IBD	Human	+	[11]
CRC	Human	+	[12–15]
	Mice or rat	+	[16–19]
	Mice	-	[20]
Celiac disease	Human	-	[21,22]
Constipation	Mice	-	[23–25]
Clostridium difficile infection	Human	+	[26–30]
	Human	-	[31]
Salmonella Typhimurium infection	Mice	+	[32–35]
Escherichia coli infection	Rat	-	[36]

Reference

1. Ponce-Alonso, M. *et al.* An Immunologic Compatibility Testing Was Not Useful for Donor Selection in Fecal Microbiota Transplantation for Ulcerative Colitis. *Frontiers in immunology* **12**, 683387 (2021).
2. Zhang, T. *et al.* Alterations of Akkermansia muciniphila in the inflammatory bowel disease patients with washed microbiota transplantation. *Applied microbiology and biotechnology* **104**, 10203–10215 (2020).
3. Earley, H. *et al.* The abundance of Akkermansia muciniphila and its relationship with sulphated colonic mucins in health and ulcerative colitis. *Scientific reports* **9**, 15683 (2019).
4. He, C. *et al.* Characteristics of mucosa-associated gut microbiota during treatment in Crohn's disease. *World journal of gastroenterology* **25**, 2204–2216 (2019).
5. Magro, D. O. *et al.* Remission in Crohn's disease is accompanied by alterations in the gut microbiota and mucins production. *Scientific reports* **9**, 13263 (2019).
6. Lopez-Siles, M. *et al.* Alterations in the Abundance and Co-occurrence of Akkermansia muciniphila and Faecalibacterium prausnitzii in the Colonic Mucosa of Inflammatory Bowel Disease Subjects. *Frontiers in cellular and infection microbiology* **8**, 281 (2018).
7. Roche-Lima, A. *et al.* The Presence of Genotoxic and/or Pro-inflammatory Bacterial Genes in Gut Metagenomic Databases and Their Possible Link With Inflammatory Bowel Diseases. *Frontiers in genetics* **9**, 116 (2018).
8. Bajer, L. *et al.* Distinct gut microbiota profiles in patients with primary sclerosing cholangitis and ulcerative colitis. *World journal of gastroenterology* **23**, 4548–4558 (2017).
9. Dunn, K. A. *et al.* Early Changes in Microbial Community Structure Are Associated with Sustained Remission After Nutritional Treatment of Pediatric Crohn's Disease. *Inflammatory bowel diseases* **22**, 2853–2862 (2016).
10. Rajilić-Stojanović, M., Shanahan, F., Guarner, F. & Vos, W. M. de. Phylogenetic analysis of dysbiosis in ulcerative colitis during remission. *Inflammatory bowel diseases* **19**, 481–488 (2013).
11. Danilova, N. A. *et al.* Markers of dysbiosis in patients with ulcerative colitis and Crohn's disease. *Terapevticheskii arkhiv* **91**, 17–24 (2019).

12. Osman, M. A. *et al.* Parvimonas micra, Peptostreptococcus stomatis, Fusobacterium nucleatum and Akkermansia muciniphila as a four-bacteria biomarker panel of colorectal cancer. *Scientific reports* **11**, 2925 (2021).
13. Campisciano, G. *et al.* The Obesity-Related Gut Bacterial and Viral Dysbiosis Can Impact the Risk of Colon Cancer Development. *Microorganisms* **8**, 431 (2020).
14. Han, S. *et al.* Adequate Lymph Node Assessments and Investigation of Gut Microorganisms and Microbial Metabolites in Colorectal Cancer. *OncoTargets and therapy* **13**, 1893–1906 (2020).
15. Sheng, Q.-S. *et al.* Comparison of Gut Microbiome in Human Colorectal Cancer in Paired Tumor and Adjacent Normal Tissues. *OncoTargets and therapy* **13**, 635–646 (2020).
16. Song, C.-H. *et al.* Changes in Microbial Community Composition Related to Sex and Colon Cancer by Nrf2 Knockout. *Frontiers in cellular and infection microbiology* **11**, 636808 (2021).
17. Lang, M. *et al.* Crypt residing bacteria and proximal colonic carcinogenesis in a mouse model of Lynch syndrome. *International journal of cancer* **147**, 2316–2326 (2020).
18. Wu, M. *et al.* The Dynamic Changes of Gut Microbiota in Muc2 Deficient Mice. *International journal of molecular sciences* **19**, 2809 (2018).
19. Xiao, X. *et al.* Differences Between the Intestinal Lumen Microbiota of Aberrant Crypt Foci (ACF)-Bearing and Non-bearing Rats. *Digestive diseases and sciences* **63**, 2923–2929 (2018).
20. Wang, L. *et al.* A purified membrane protein from Akkermansia muciniphila or the pasteurised bacterium blunts colitis associated tumourigenesis by modulation of CD8(+) T cells in mice. *Gut* **69**, 1988–1997 (2020).
21. Di Biase, A. R. *et al.* Gut microbiota signatures and clinical manifestations in celiac disease children at onset: a pilot study. *Journal of gastroenterology and hepatology* **36**, 446–454 (2021).
22. Bodkhe, R. *et al.* Comparison of Small Gut and Whole Gut Microbiota of First-Degree Relatives With Adult Celiac Disease Patients and Controls. *Frontiers in microbiology* **10**, 164 (2019).
23. Kim, M. G. *et al.* Prebiotics/Probiotics Mixture Induced Changes in Cecal Microbiome and Intestinal Morphology Alleviated the Loperamide-Induced Constipation in Rat. *Food science of animal resources* **41**, 527–541 (2021).
24. Ma, H. *et al.* Polysaccharide from Spirulina platensis ameliorates diphenoxylate-induced constipation symptoms in mice. *International journal of biological macromolecules* **133**, 1090–1101 (2019).
25. Yi, R. *et al.* Lactobacillus plantarum CQPC02-Fermented Soybean Milk Improves Loperamide-Induced Constipation in Mice. *Journal of medicinal food* **22**, 1208–1221 (2019).
26. Vakili, B., Fateh, A., Asadzadeh Aghdaei, H., Sotoodehnejadnematalahi, F. & Siadat, S. D. Characterization of Gut Microbiota in Hospitalized Patients with Clostridioides difficile Infection. *Current microbiology* **77**, 1673–1680 (2020).
27. Vakili, B., Fateh, A., Asadzadeh Aghdaei, H., Sotoodehnejadnematalahi, F. & Siadat, S. D. Intestinal Microbiota in Elderly Inpatients with Clostridioides difficile Infection. *Infection and drug resistance* **13**, 2723–2731 (2020).
28. Araos, R. *et al.* Fecal Microbiome Among Nursing Home Residents with Advanced Dementia and Clostridium difficile. *Digestive diseases and sciences* **63**, 1525–1531 (2018).

29. Hernández, M. *et al.* Fecal Microbiota of Toxigenic Clostridioides difficile-Associated Diarrhea. *Frontiers in microbiology* **9**, 3331 (2018).
30. Sangster, W. *et al.* Bacterial and Fungal Microbiota Changes Distinguish C. difficile Infection from Other Forms of Diarrhea: Results of a Prospective Inpatient Study. *Frontiers in microbiology* **7**, 789 (2016).
31. Rodriguez, C. *et al.* Longitudinal survey of Clostridium difficile presence and gut microbiota composition in a Belgian nursing home. *BMC microbiology* **16**, 229 (2016).
32. Wang, R. *et al.* Protective Effects of Cinnamaldehyde on the Inflammatory Response, Oxidative Stress, and Apoptosis in Liver of Salmonella typhimurium-Challenged Mice. *Molecules (Basel, Switzerland)* **26**, 2309 (2021).
33. Hao, S. *et al.* Core Fucosylation of Intestinal Epithelial Cells Protects Against Salmonella Typhi Infection via Up-Regulating the Biological Antagonism of Intestinal Microbiota. *Frontiers in microbiology* **11**, 1097 (2020).
34. Xu, X. *et al.* Glycyrrhizin Attenuates Salmonella enterica Serovar Typhimurium Infection: New Insights Into Its Protective Mechanism. *Frontiers in immunology* **9**, 2321 (2018).
35. Borton, M. A. *et al.* Chemical and pathogen-induced inflammation disrupt the murine intestinal microbiome. *Microbiome* **5**, 47 (2017).
36. Sun, X. *et al.* Escherichia coli O(101)-induced diarrhea develops gut microbial dysbiosis in rats. *Experimental and therapeutic medicine* **17**, 824–834 (2019).
